# Assessing Ureteral Patency by Fluoroscopy and Ultrasonography After Subcutaneous Ureteral Bypass Device Placement for the Treatment of Benign Ureteral Obstruction in Cats

**DOI:** 10.1111/jvim.70078

**Published:** 2025-04-28

**Authors:** Yassmina Habib, Catherine Vachon, Tristan Juette, Marilyn Dunn

**Affiliations:** ^1^ Department of Clinical Sciences, Cummings School of Veterinary Medicine Tufts University North Grafton Massachusetts USA; ^2^ Department of Clinical Sciences, School of Veterinary Medicine University of Montreal Saint‐Hyacinthe Quebec Canada

**Keywords:** calcium oxalate, interventional radiology, pyelogram, ureteral obstruction, ureteral stricture, ureteroliths, urinary obstruction

## Abstract

**Background:**

Limited information on the patency of native ureters after subcutaneous ureteral bypass (SUB) device placement is available.

**Objective:**

Evaluate the patency of native ureters in cats treated with SUB device placement for benign ureteral obstruction.

**Animals:**

Cats with SUB presented for routine evaluation.

**Methods:**

Routine ultrasound‐guided SUB irrigations were performed, focusing on ureteral irrigation in the trigone and proximal urethra. Cats with obstructed nephrostomy catheters or subcutaneous ports were excluded. Fluoroscopic‐guided SUB irrigations with iodinated contrast then were used to assess patency. Ureters were deemed patent if contrast filled the lumen evenly along their length, and obstructed if the contrast column was interrupted on consecutive images. Intraoperative fluoroscopy was reviewed to confirm the cause and location of the obstructions.

**Results:**

Overall, 18 cats (18 SUBs; 10 unilateral, 8 bilateral) were included. The causes of obstruction were ureteroliths (23 ureters) and presumed stricture (3 ureters). A trigonal irrigation was visible in 14/18 cats (77%; 95% confidence interval [CI]: 54.8%–91.0%); the patent side in bilateral SUBs could not be differentiated. Three ureters were excluded (nondiagnostic study, *n* = 1; obstructed nephrostomy, *n* = 2). Of the remaining 23 ureters, 19 (82.6%; 95% CI: 62.9%–93.0%) were patent: 16/20 patent ureters were obstructed with ureteroliths (80%; 95% CI: 58.4%–91.9%) and 3/3 with presumed stricture (100%; 95% CI: 43.9%–100%). Ureteral irrigations at the trigone were associated with patency.

**Conclusions:**

A high patency rate of native ureters after SUB placement was observed. Ureteral irrigations at the trigone reliably indicate patency. Supraphysiologic SUB irrigations may result in underdiagnosed persistent partial obstructions.

**Abbreviations**
CKD: chronic kidney disease
FUO: benign ureteral obstructionin cats
SUB: subcutaneous Ureteral Bypass
tEDTA: tetrasodium ethylenediaminetetraacetic acid
US: ultrasonography

## 1 Introduction

Ureteral obstruction in cats (FUO) occurs for several reasons, including ureterolithiasis, ureteral stricture, pyonephrosis, and iatrogenic ureteral ligation [1, 2, 3, 4, 5, 6, 7, 8, 9, 10, 11, 12, 13, 14, 15, 16, 17, 18]. At presentation, affected cats are often azotemic, and the majority have concurrent kidney disease (CKD) [5, 15, 19, 20, 21, 22, 23, 24]. Ureterolithiasis is the most common cause of FUO [8, 20, 21, 25]. A study of ureteral calculi in cats found that 98% were composed of calcium oxalate [5]. Response to medical management in cats with FUO has been described as poor [26]. Improved outcomes recently have been reported in 72 cats undergoing medical management, with an overall success rate of 30% [27].

Surgical management of FUO includes ureterotomy, ureteral resection and anastomosis, and subcutaneous bypass device (SUB) placement (Norfolk Veterinary Products) [20, 26, 28, 29]. Subcutaneous ureteral bypass device placement has been extensively studied, is associated with an excellent prognosis, and has become the standard approach for treatment of FUO in many institutions [20, 29, 30, 31, 32]. The devices are equipped with multifenestrated pigtail catheters that are placed in the renal pelvis and bladder, allowing urine to flow from the renal pelvis to the bladder and bypass the obstructed ureter. The subcutaneous port provides access for urine sampling, and saline or an iodinated contrast agent can be infused to confirm patency of the system [33]. Subcutaneous ureteral bypass devices are effective in relieving ureteral obstruction in cats, regardless of the underlying cause [18, 20, 34, 35, 36, 37, 38, 39]. The most common long‐term complication associated with SUB placement is luminal obstruction from mineralization. Subcutaneous ureteral bypass device obstruction is suspected when evidence of pyelectasia is observed on ultrasonography, and when ultrasound‐guided irrigation cannot be visualized in the kidney, the bladder, or both. This complication is reported in 17%–24% of implanted devices [20, 32]. However, only 6%–12.7% of such devices required replacement because ureteral obstruction was not present despite obstruction of the SUB. Reestablishment of ureteral patency was suspected [20, 32]. In partially obstructed SUBs, infusion of tetrasodium ethylenediaminetetraacetic acid (tEDTA) through the subcutaneous port resulted in successful demineralization and restored patency in mineralized SUBs [40, 41]. In cats with completely obstructed SUBs or those refractory to tEDTA infusion with renal pelvic or ureteral dilatation or both, surgical catheter exchange is indicated. Ureteral patency can be difficult to assess in a cat with a patent SUB. In cats with an obstructed cystostomy catheter without evidence of pelvic dilatation, we have visualized ultrasound‐guided saline irrigation at the trigone or proximal urethra, supporting native ureteral patency. Documenting ureteral patency in patients with SUBs could help guide clinical decisions, such as determining the urgency to perform SUB revision surgery in the case of SUB catheter obstruction, and provide insight into the pathophysiology of ureteral obstruction in cats.

Results of fluoroscopically‐guided SUB irrigation are less commonly reported, and at our institution, this procedure is used primarily when SUB migration or obstruction is suspected after a challenging ultrasound‐guided irrigation (i.e., resistance during irrigation, inability to sample urine through the port, poor visualization of irrigation though the nephrostomy or cystotomy catheters or both). Native ureteral patency after SUB placement has not been thoroughly investigated. A recent study described postoperative patency in 92/115 bypassed ureters using fluoroscopically‐guided SUB device irrigation. The study reported that 54% of ureters assessed were patent, 94% of which remained patent over time [33]. Factors associated with patency were not reported. Furthermore, a previous study reported that temporary ureteral stenting by percutaneous nephrostomy successfully relieved experimentally induced ureteral obstruction in dogs, resulting in long‐term resolution of obstruction compared with untreated controls [42].

Our objectives were to: (1) document ureteral patency in cats with SUBs placed for the treatment of FUO; (2) evaluate the rate of ureteral patency after SUB placement for various causes of FUO (cause of obstruction determined by ultrasonography and intra‐operative antegrade ureteropyelography during SUB placement); (3) document findings during ultrasound‐guided SUB irrigations that indicate ureteral patency; and (4) compare ultrasound‐guided SUB irrigation findings to the results of fluoroscopically‐guided SUB irrigations (Video 1).

**VIDEO 1:** Ultrasound‐guided SUB flush of a cat with an obstructed cystotomy catheter. Sterile saline is injected through the subcutaneous port. Flush bubbling from the cystotomy catheter at the bladder apex and trigonal flush originating from the bladder trigone is observed. Video content can be viewed at https://onlinelibrary.wiley.com/doi/10.1111/jvim.70078

## 2 Materials and Methods

Cats with SUBs placed for FUO presented to the Centre hospitalier universitaire vétérinaire (CHUV) of the University of Montreal for routine evaluation and SUB irrigation were recruited. Informed owner consent to participate in the study was obtained. Information retrieved from the medical records included signalment, cause of the ureteral obstruction, CBC, serum biochemistry including serum ionized calcium concentration, and urine bacterial culture as well as the date of SUB placement. Data collected on follow‐up examinations included clinical history, presence or absence of lower urinary tract signs, hematocrit, serum phosphorus, creatinine, ionized calcium, and SDMA concentrations, urine bacterial culture results, and patency of the SUB after ultrasound‐guided irrigation and type of locking solution used (saline or tEDTA).

**FIGURE 1:**
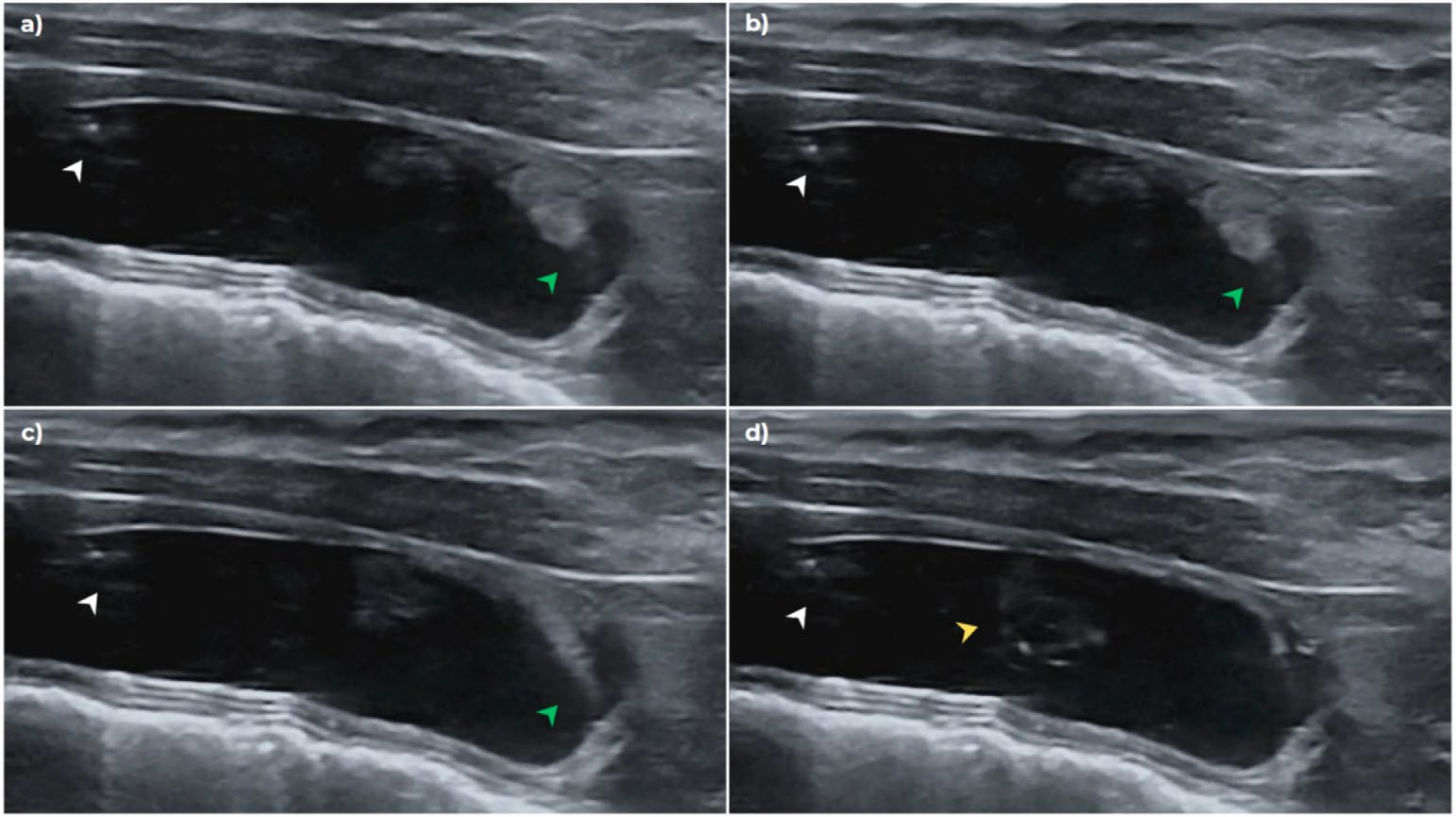
Ultrasound images of a SUB irrigation. Sterile saline is injected through the subcutaneous port. (a) Irrigation bubbling from the cystotomy catheter at the bladder apex (white arrow) and trigonal irrigation originating from the bladder trigone (green arrow); (b) similar bubbling from the cystotomy catheter while trigonal irrigation continues to be seen; (c) trigonal irrigation into the bladder while bubbling at the bladder apex stops; and (d) trigonal irrigation spirals in the urinary bladder (yellow arrow).

Ultrasound‐guided SUB irrigation was performed routinely. Briefly, cats were placed in dorsal recumbency, and the skin over the port was shaved and aseptically prepared. A 22 G 3/4″ Huber needle was connected to a 12″ extension set, a 3‐way stopcock (Smiths medical) and two 5‐mL syringes were connected; one with 3 mL of saline and the other empty. Upon penetrating the port with the Huber needle, 1.0–3.0 mL of urine was withdrawn and saved for culture for SUB 2.0 models, and then the irrigation was performed by a rapid injection of 0.5–1.0 mL aliquots of sterile 0.9% sodium chloride into the port while visualizing the kidney on ultrasonography. Between 0.5 and1.0 mL then was withdrawn from the port, and an additional 0.5 mL of saline was injected rapidly while visualizing the bladder on ultrasonography. For SUB 3.0 models, 1 mL of urine was discarded before sampling for culture. Cats were determined to be obstructed if no irrigation could be visualized either in the kidney, bladder, or both. The bladder trigone and proximal urethra were carefully observed during irrigation for jets not arising from the cystostomy catheter at the bladder apex (trigone irrigation; Figure 1). The ultrasound‐guided irrigation served as an initial screening for SUB patency. Cats with an obstructed nephrostomy catheter, subcutaneous port, or both were excluded from the study. Cats with a bilateral SUB and obstruction of a single nephrostomy catheter were included in the study, but only results for the patent nephrostomy catheter and accompanying ureter were reported. Cats that were too fractious to be handled or medically unstable also were excluded.

**FIGURE 2:**
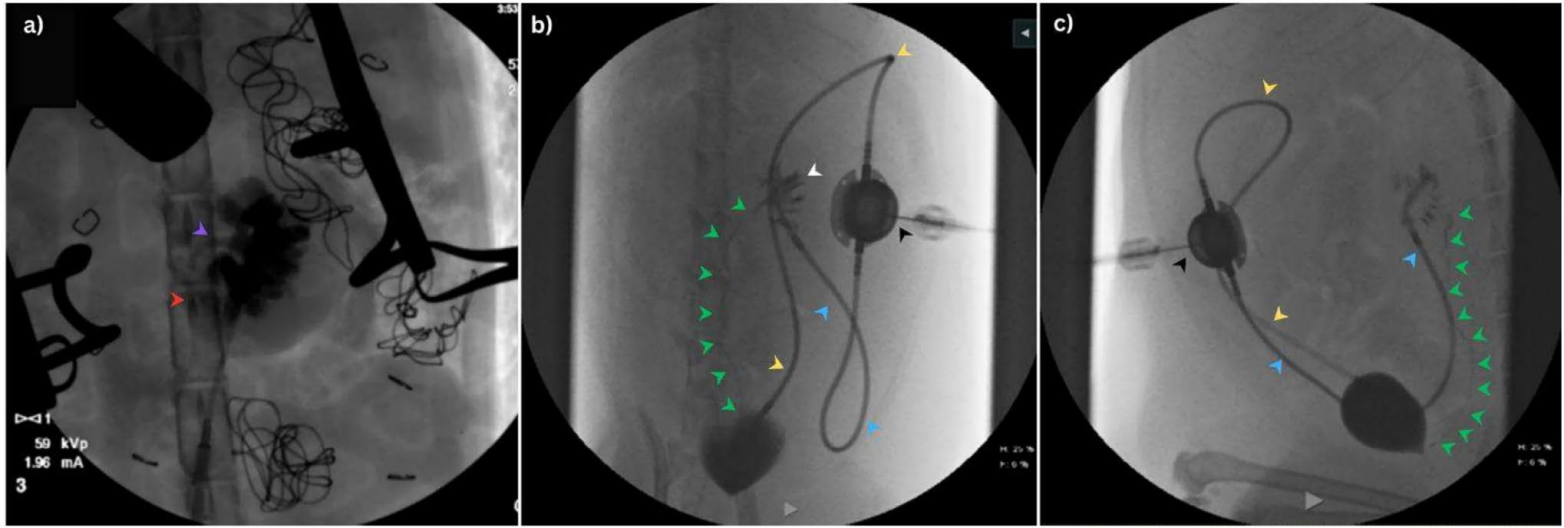
Patient with a unilateral SUB 2.0 with regained ureteral patency. (a) Intraoperative fluoroscopic antegrade ureteropyelogram of the patient showing contrast filling a markedly dilated renal pelvis. The proximal ureter is dilated (purple arrow). The contrast column abruptly ends at the proximal (red arrow). Distally, the ureter is not filling with contrast, demonstrating a complete obstruction. (b and c) The same patient in dorsal (b) and lateral recumbency (c) during fluoroscopy 2 years after SUB placement. Fluoroscopic study shows contrast injection through the subcutaneous port (black arrow). Contrast can be seen filling the nephrostomy catheter (blue arrow) and the cystostomy catheter (yellow arrow). The renal pelvis (white arrow) is filled with contrast; the bypassed ureter is filled with contrast along its entire course (green arrows).

**FIGURE 3:**
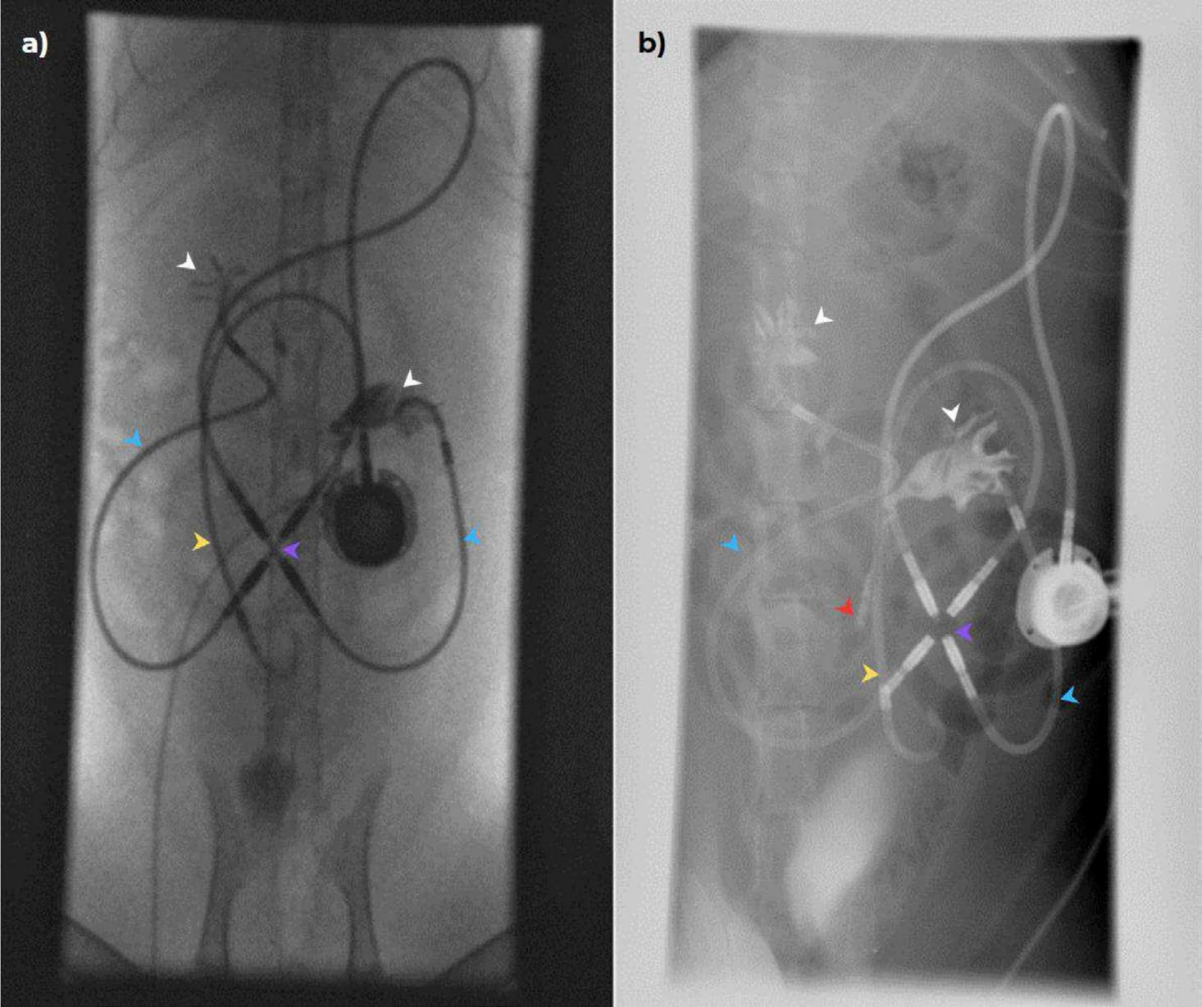
A cat with a bilateral SUB 3.0 (X connector identified by purple arrow) with obstructed ureters. (a) Nephrostomy (blue arrow) and cystotomy catheters (yellow arrow) were confirmed as patent. Filling of both renal pelves with contrast was achieved (white arrow), however the entire courses of the ureters were not observed on fluoroscopy; (b) the patient was placed oblique position and digital radiography confirmed persistent ureteral obstruction bilaterally (contrast in the left ureter abruptly stops as indicated by the red arrow). The patient imaged in this study previously had a SUB 2.0 model, which was converted into a 3.0 model due to catheter kinking causing repeated SUB occlusions. An X connector was placed during the conversion to the 3.0 model. The SUB 2.0 cystotomy catheter remains as part of the imaged SUB device.

**FIGURE 4:**
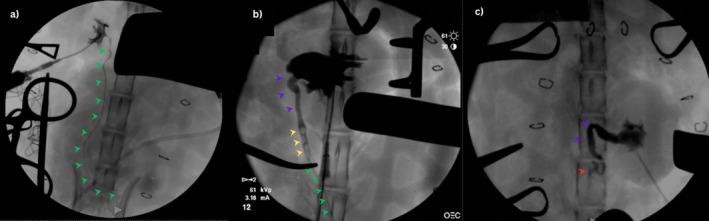
Intraoperative fluoroscopic antegrade ureteropyelograms in cats showing normal ureteral patency (a), partial ureteral obstruction, (b) and complete ureteral obstruction (c). (a) Contrast can be seen filling the renal pelvis and the ureter (green arrow) is filling with contrast evenly over it is entire course to the urinary bladder. (b) Contrast can be seen filling the severely dilated renal pelvis. The proximal ureter is tortuous and dilated (purple arrow) proximally to three ureteral stones (yellow arrow). Distally to the ureteral stones, the ureter (green arrow) is evenly filling with contrast and is considered normal in size, demonstrating a partially obstructed ureter. (c) Contrast can be seen filling the dilated renal pelvis. The proximal ureter is tortuous and dilated (purple arrow) until abrupt end (red arrow). Distally, the ureter is not filling with contrast, demonstrating a completely obstructed ureter.

Overall, 10 cats routinely received gabapentin (Summit Veterinary Pharmacy; 20–50 mg PO) before veterinary consultation and sometimes required further sedation for the SUB irrigation with 0.2–0.3 mg/kg butorphanol (Torbugesic, Zoetis‐CA) IM. The fractious cats received 0.3 mg/kg of butorphanol IM with 5 μg/kg dexmedetomidine (Dexdomitor, Zoetis‐CA) IM or 1 mg/kg alfaxolone (Alfaxan Multidose, Zoetis‐CA) IM. After ultrasound‐guided SUB irrigation, the cat was taken to the fluoroscopy room, placed in dorsal recumbency, and after sterile preparation of the port site, a Huber needle connected to an extension set as described above was used to drain urine from the system. Overall, 8–10 mL of undiluted iohexol (Isovue, Bracco Diagnostics) was infused during approximately 20–30 s under fluoroscopic guidance to obtain filling of the renal pelvis and ureter. A combination of standard fluoroscopy, digital subtraction (DS) and dynamic radiology (DR) was performed. To identify the entire course of the ureter from the kidney to its entry into the trigone, cats occasionally were rotated (oblique) or placed in lateral recumbency as needed to allow assessment of the ureter along the entirety of its course. Under fluoroscopic guidance, at the discretion of the attending clinician, 1–3 mL of tEDTA or saline then were injected through the subcutaneous port under fluoroscopic guidance to serve as a locking solution and avoid overfilling of the pelvis. Ureters were described as patent if contrast evenly filled the lumen along its entire length (Figure 2) or obstructed if the column of contrast stopped or was interrupted on consecutive images (Figure 3). A ureter was classified as patent when its entire path could be followed on fluoroscopy, and the lumen was evenly filled with contrast throughout; otherwise, it was classified as “not patent.” Intra‐operative fluoroscopic images were reviewed to confirm the cause and location of obstruction and to assess for previous pelvic or ureteral dilatation (Figure 4).

Sensitivity and specificity of the observation of the trigonal irrigation on ultrasonography in assessing ureteral patency were calculated using fluoroscopy data of patency as the gold standard. Calculations of 95% confidence interval (95% CI) for proportions were calculated using the Wilson score method to provide adequate estimates for small sample sizes and extreme proportions. Calculations of 95% CI related to specificity and sensitivity was performed using the Clopper‐Pearson method, suitable for small samples (*n* < 30). Furthermore, serum creatinine concentrations at the time of the study in cats with obstructed versus patent ureters on fluoroscopy were compared.

## 3 Results

Overall, 20 cats initially were enrolled during the study period (February 2022—October 2023). The mean duration between SUB placement and enrollment in the study was 1 year and 1 month (range, 1 month to 6 years and 2 months). Two cats with unilateral SUBs were excluded because of undiagnostic fluoroscopic studies. Nine neutered male and nine spayed female cats were included in the study with a median age of 8.7 years (range, 3.7–14 years) and median weight of 5.05 kg (range, 3.31—6.96 kg). Overall, 13 cats were domestic shorthair or longhair, and five were purebreds (Himalayan, *n* = 1; Persian, *n* = 1; Russian Blue, *n* = 1; Abyssinian, *n* = 1; Burmese, *n* = 1). Median time from SUB placement to enrollment in the study was 1 year (range, 1 month to 6 years and 2 months). Each cat enrolled was only imaged once. Overall, 18 SUBs in our study included 10 unilateral SUBs and 8 bilateral SUBs. Models 2.0 (*n* = 7) and 3.0 with an X connector (*n* = 11) were represented. The cause of obstruction of the 26 bypassed ureters included ureteroliths (*n* = 23) and presumed ureteral strictures (*n* = 3).

At the time of SUB placement, the median serum creatinine concentration was 193 μmol/L (range, 141–1861 μmol/L), median renal pelvis size on ultrasonography was 13 mm (range, 5–24 mm) and median ureteral diameter was 4 mm (range, 1.0–7.0 mm). At the time of the fluoroscopic examination, the mean serum creatinine concentration was 208 μmol/L (range, 116–358 μmol/L), mean renal pelvis size on ultrasonography was 0.5 mm (range, 0–6 mm) and mean ureteral diameter was 1 mm (range, 0–2.3 mm).

### 3.1 Ultrasound‐Guided SUB Irrigation

Saline irrigation was visualized originating from the bladder trigone or proximal urethra (trigone irrigation) in 14/18 (77%) cats (95% CI = 54.8%–91.0%). All cats in which a trigone irrigation was seen had a patent ureter on fluoroscopy. Two of 18 (11%) cats with bilateral SUBs were suspected on ultrasonography to have one obstructed nephrostomy catheter each, which then was confirmed as obstructed on fluoroscopy; the two corresponding bypassed ureters were excluded from further analyses (*n* = 24 ureters). The two ureters excluded were previously documented to be obstructed by ureteroliths.

### 3.2 Fluoroscopic‐Guided SUB Irrigation

Overall, 24 ureters were evaluated by fluoroscopy. Filling of both renal pelves and achieving a diagnostic study bilaterally was more challenging in cats with bilateral SUB 3.0. Filling of the bladder and one renal pelvis tended to occur, and multiple injections and aspirations were required to correctly fill both renal pelves in some cats. After review of the fluoroscopic images, the study was evaluated as non‐diagnostic for one additional ureter because of poor renal pelvis filling (one ureter bypassed by a bilateral SUB 3.0). The ureter excluded initially was diagnosed as obstructed by ureteroliths. Of the 23 ureters that could be adequately assessed by fluoroscopy (appropriate contrast filling of the renal pelvis), 19 bypassed ureters were classified as patent (19/23, 82.6%; 95% CI = 62.9%–93.0%). Overall, 16 patent ureters previously were documented as obstructed by ureteroliths (16/20, 80%; 95% CI = 58.4%–91.9%). Three patent ureters were previously documented as obstructed by presumed stricture (3/3, 100%; 95% CI = 43.9%–100%). To visualize the entire course of the distal ureter, oblique fluoroscopic views were required in 17/23 (74%) ureters (95% CI = 53.5%–87.5%) and digital subtraction was performed in 2/23 (8.7%) ureters (95% CI = 2.4%–26.8%). The sensitivity of ultrasonographic trigone imaging in assessing ureteral patency was 87.5% (16/18 cats; 95% CI = 61.7%–98.5%) whereas the specificity was 100.00% (2/2 cats; 95% CI: 15.8%–100.0%).

## 4 Discussion

We studied ureteral patency in cats after SUB placement. Overall, 19 of 23 (82.6%) ureters evaluated were patent on fluoroscopy, ranging from 1 month to 6 years after surgery. This patency rate is higher than previously reported in cats [33]. This study also included cats with at least one patent nephrostomy catheter and therefore, as in our study, patency could not be assessed in some cats. Interestingly, cats in our study excluded because of an obstructed nephrostomy catheter did not have signs of ureteral obstruction (dilated renal pelvis, dilated ureter, increased serum creatinine concentration), which may suggest a patent or partially patent ureter. Furthermore, observation of a trigone irrigation during routine ultrasound‐guided SUB irrigation is a reliable indicator of ureteral patency, with a specificity of 100% and sensitivity of 87.5%.

Our study raises the question as to whether SUB placement directly contributed to the re‐establishment of ureteral patency or whether ureteral obstruction is transitory and resolves in some cats. An experimental model of ureteral obstruction in dogs demonstrated calculi passage and resolution of obstruction after urinary tract decompression [42]. Two recent studies have reported ureterolith passage rates of 23%–31.4% [27, 43] and resolution of ureteral obstruction secondary to stricture in 50% of cats undergoing medical management [27]. Cats undergoing SUB placement receive fluids, antibiotics, analgesics, and occasionally anti‐spasmodic drugs pre‐ and post‐operatively, similar to cats that underwent medical management in the studies cited above. Bypassing the obstructed ureter may relieve distention of the renal pelvis and ureter, both of which are associated with ureteral colic in people [44]. The resulting spasm and inflammation may contribute to ureteral obstruction and explain the high rate of ureteral patency after relief of obstruction by SUB placement.

The three cats diagnosed with presumed strictures resulting in ureteral obstruction and SUB placement all had patent ureters on fluoroscopy. Contrast was seen passing through the previously obstructed site on fluoroscopy in a uniform manner without evidence of ureteral dilatation. This finding was surprising because it has been assumed that strictures may be the result of ureteral fibrosis, but such was not the case in these three cats. Resolution of ureteral obstruction after medical management of presumed ureteral strictures has been reported [27]. Histopathology of presumed strictures would be useful to better understand these situations in cats.

Several challenges were encountered during the fluoroscopic portion of the study. Bladder position and size obscured visualization of the distal ureter in some cats. Emptying the bladder completely before performing the study and placing the cat in an oblique position or lateral recumbency or both improved evaluation of ureteral patency. In cats with full colons or caudally positioned bladders, digital subtraction aided in visualization of the distal ureter. Cats with bilateral ureteral obstruction and a SUB 3.0 were more challenging to image. Bilateral SUB 3.0 all had x‐connectors linked to one cystostomy catheter. One renal pelvis was more difficult to fill than the bladder and contralateral pelvis in some cats. We believe this difficulty was caused by the path of least resistance being firstly the bladder (expected) and then one of two kidneys, the parenchyma of which may have been easier to compress with the supraphysiologic pressure exerted by irrigation. For contrast to reach the renal pelvis and then travel through a patent ureter, the column of urine must first be displaced. Unequally decreased compliance and, therefore, increased resistance in a renal pelvis may explain this challenge.

Our study evaluated ureteral patency based on supraphysiologic irrigation of a contrast agent into the renal pelvis with subsequent ureteral flow. It is possible that partial obstructions persisted but were missed or underestimated given the supraphysiologic pressure and large amount of contrast agent administered to fill the renal pelvis and evaluate flow through the ureter. Normal urine output in cats is approximately 2 mL/kg/h. The median weight of cats in our study was 5.1 kg. Therefore, a 5.1 kg cat would be expected to produce 10.2 mL of urine per hour or 0.0028 mL/s or 0.0014 mL/s/kidney. A typical ultrasound‐guided irrigation lasts 2–3 s, which amounts to urine production of 0.0028–0.0042 mL/kidney. Knowing that the irrigations in our study ranged from 1 to 3 mL/irrigation, these values alone demonstrate that a 3‐s irrigation represents in volume 238–1071 times the normal urinary output per kidney. Over the average 5–10‐min period needed to perform a fluoroscopic SUB irrigation, cats received approximately 6–8 mL of irrigation solution. During a 5–10‐min period, the kidney normally produces between 0.42–0.84 mL of urine, which again emphasizes that irrigation volumes were 7.1–19 times higher than physiological urinary output.

To our knowledge, our study represents the first time that ultrasonographic observation of a trigone irrigation has been evaluated as an indicator of ureteral patency in cats with SUBs. Trigone irrigation was an indicator of some ureteral patency with a specificity of 100% and sensitivity of 87.5%. However, trigonal irrigation only indicates that there is some ureteral flow through the native ureter. Given the supraphysiologic pressure and volume when irrigating, trigonal irrigation could be observed with a partial ureteral obstruction.

Assessing trigonal irrigation during ultrasound‐guided SUB irrigations is an easy method to confirm ureteral patency and does not necessitate additional manipulations or radiation exposure. However, similar to the fluoroscopic study, there are some limitations to its interpretation. Visualization of a trigonal irrigation in cats with a bilateral SUB can only confirm the patency of one ureter. It was not possible in these cats to evaluate, which ureter was patent based on the presence of a trigonal irrigation. Patient positioning and bladder size also were limiting factors.

Our study had some limitations. Cats with SUBs only underwent fluoroscopic imaging once at variable times after SUB placement (1 month to 6 years). It is therefore not possible to determine when the ureters regained patency or for how long patency was maintained. A previous study reported that of 32 ureters that were imaged at multiple time points, 30 maintained patency over time [33]. Given the small number of cats evaluated and only three cats with presumed ureteral stricture as the cause of their obstruction, it is not possible to speculate about whether ureteral patency is more likely to be achieved in cats with ureteroliths or presumed stricture.

In conclusion, we report a high rate of ureteral patency in cats having undergone SUB placement. Trigone irrigation was assessed as a reliable indicator of ureteral patency. Furthermore, assessing trigonal irrigation during ultrasound‐guided SUB irrigations is an easy method to confirm ureteral patency without the need for additional manipulation or radiation exposure.

## Disclosure

Authors declare no off‐label use of antimicrobials.

## Ethics Statement

Approved by the Ethics and Animal Welfare Committee (CÉUA) of the University of Montreal (21‐Rech‐2167). Authors declare human ethics approval was not needed.

## Conflicts of Interest

The authors declare no conflicts of interest.
